# Dynamic functional characterization and phylogenetic changes due to Long Chain Fatty Acids pulses in biogas reactors

**DOI:** 10.1038/srep28810

**Published:** 2016-06-29

**Authors:** Panagiotis G. Kougias, Laura Treu, Stefano Campanaro, Xinyu Zhu, Irini Angelidaki

**Affiliations:** 1 Department of Environmental Engineering, Technical University of Denmark, Kgs. Lyngby, Denmark; 2 Department of Biology, University of Padova, Via U. Bassi 58/b, 35121, Padova Italy

## Abstract

The process stability of biogas plants is often deteriorated by the accumulation of Long Chain Fatty Acids (LCFA). The microbial community shifts due to LCFA disturbances have been poorly understood as the molecular techniques used were not able to identify the genome characteristics of uncultured microorganisms, and additionally, the presence of limited number of reference genomes in public databases prevented the comprehension of specific functional roles characterizing these microorganisms. The present study is the first research which deciphers by means of high throughput shotgun sequencing the dynamics of the microbial community during an inhibitory shock load induced by single pulses of unsaturated LCFA at two different concentrations (i.e. 2 g/L-reactor and 3 g/L-reactor). The metagenomic analysis showed that only the microbes associated with LCFA degradation could encode proteins related to “chemotaxis” and “flagellar assembly”, which promoted the ability to move towards the LCFA sources so as to degrade them. Moreover, the syntrophic interactions found between *Syntrophomonas* sp. together with *Methanosarcina* sp. were possibly assigned to the menaquinone-electron transfer. Finally, it was proven that a previously exposed to LCFA inoculum is more efficient in the degradation process of LCFA due to the specialization of the microbial consortium.

Anaerobic digestion is a striking biological mediated process to treat organic residues and produce energy in the form of methane. Biogas plants apply co-digestion strategies to maximize the energy yield and achieve a cost-sharing process by treating multiple waste streams in a single facility^1^. Practically, this means that the mixture of the influent feedstock varies during the year, depending on the availability of the seasonal biomass. As a consequence, the concentration of specific compounds (e.g. ammonia load, fatty acids etc) may suddenly increase above a critical threshold and therefore act as inhibitors, causing imbalances to the anaerobic digestion (AD) process. Therefore, it is of major importance to monitor the changes in the chemical composition of the influent feedstock in order to ensure stable reactor operation. A frequent and common upset is caused during the overload of biogas reactors with lipid rich substrates, which might result in accumulation of Long Chain Fatty Acids (LCFA).

LCFA are amphiphilic compounds produced via the rapid hydrolysis of lipids by extracellular lipases^2^. The anaerobic degradation of LCFA proceeds through the β-oxidation pathway, in which a repetitive cleavage of 2-carbon fragments occurs with concomitant release of acetyl-CoA. The inhibition caused by LCFA relies to cases that major products of β-oxidation accumulate to thermodynamically-limiting levels that prevent LCFA (and propionate) to be further oxidized^3^. By monitoring the operational parameters of the anaerobic digesters, the inhibition can be easily designated by a distinct lag phase during which the methane productivity is decreased^4^.

The negative effect of the LCFA accumulation on the anaerobic microbial consortium in biogas reactors is well known. It has been previously documented that LCFA could inhibit the activity of hydrolytic, acidogenic, acetogenic bacteria and methanogenic archaea^5^,^6^,^7^. However, the archaeal community was found to be more tolerant to increased LCFA concentration levels compared to the bacterial community^8^. Moreover, it was shown that the hydrogenotrophic methanogens are more resilient to the toxic effects from LCFA compared to the aceticlastic methanogens^5^,^6^,^9^,^10^. The inhibition of methanogenesis was initially proposed to be permanent and was attributed to the lipophilic properties of LCFA that permits them to be absorbed into the surface of microbial cell causing membrane lysis^11^. Nevertheless, more recent studies proved that LCFA do not exert a bactericidal effect towards methanogens and therefore the inhibition, which is rather associated with the mineralization of LCFA, is reversible^7^.

Previous researches on the microbial community composition during the LCFA degradation in biogas reactors demonstrated that there is a significant role of syntrophic association between acetogenic bacteria and methanogenic archaea or sulphate-reducing bacteria^12^. This behavior is attributed to the unfavorable energetic balance during the degradation process of LCFA, which obliges the bacteria to uptake energy for growth, only when the concentration of the metabolic intermediates (hydrogen and/or formate) is maintained at low levels^13^. It has been reported that the main bacterial group involved in the LCFA degradation belong to the families *Clostridiaceae*, *Syntrophomonadaceae* and *Syntrophaceae*. Moreover, the main methanogenic archaea that function syntrophically with these bacteria can utilize hydrogen and belong to several genera, such as *Methanosarcina*, *Methnospirillum* and *Methanobacterium*^12^,^14^. Nevertheless, in all these studies the molecular techniques used (i.e. DGGE or 16S rRNA amplicon sequencing) were not able to provide the genome characteristics of the uncultured microorganisms that were found to increase in abundance after LCFA addition. Furthermore, the presence of a limited number of reference genomes in public databases prevents the comprehension of the specific functional roles characterizing these microorganisms. Therefore, there is an imperative need to gain a deeper insight into the microbial community profile and functions during the LCFA degradation. *De novo* assembly of shotgun sequence data followed by binning process improves the reliability of gene finding and annotation and offers the possibility to discover novel genomic elements.

The aim of this work was to decipher the dynamics of the microbial community by means of high throughput shotgun sequencing during an inhibitory shock load induced by single pulses of unsaturated LCFA at two different concentrations. Metagenomic analysis and monitoring of the most important biochemical parameters unveiled for the first time the rationale beyond the microbial community shifts occurring due to a LCFA shock. Moreover, in our study we performed a comparative analysis using inocula acclimatized and non-acclimatized to LCFA exposure, in order to elucidate the role of the microbial consortium specialized in lipid degradation. The gained knowledge will assist in better design of biogas reactors for tackling varying LCFA loads and additionally will contribute in the formulation of co-digestion strategies in full scale biogas plants.

## Results and Discussion

### Reactor performance

The injection of the LCFA pulse perturbed the stability of the process which was clearly recorded by monitoring the methane productivity of the reactors. The statistical analysis (GLM) confirmed a significant correlation (p < 0.001) between methane production and the different experimental periods (i.e. before, during and after the LCFA pulse).

**Figure 1:**
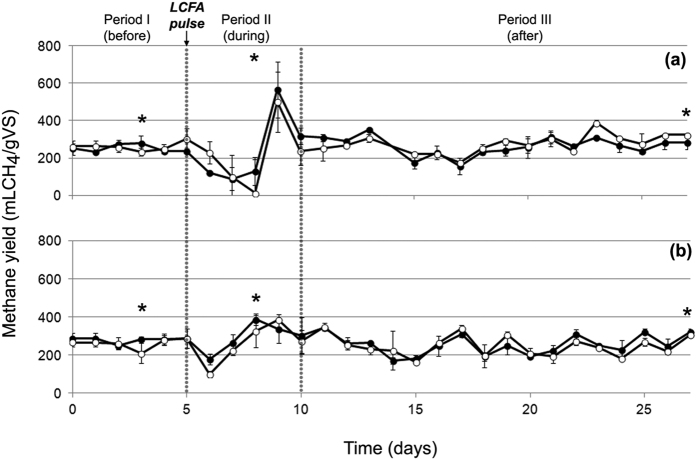
Methane yield in the reactors with (**a**) not acclimatised and (**b**) acclimatised to LCFA inoculum. During Period II, LCFA was injected as a single pulse to each reactor at concentrations of (●) 2g/L-reactor or (○) 3g/L-reactor. The asterisk symbol (*) indicates the days of DNA extraction.

In Period I, during which autoclaved manure was the explicit substrate, the methane yield did not vary significantly among reactors at both experimental sets reaching on average 260 mL CH_4_/gVS (Fig. 1). Therefore, it is clear that independent of the used inoculum the same methane yield of the manure was achieved. This is attributed to the low lipid content in cattle manure (5–8% of VS)^15^, which would not require an acclimatized to LCFA microbial consortium so as to be efficiently degraded. During period II, Na-Oleate, as a representative of unsaturated LCFA, was injected to the reactors as a single pulse at concentrations of 2 g/L-reactor and 3 g/L-reactor. The results demonstrated that the higher the concentration of the LCFA pulse, the greater the process inhibition (Fig. 1). More specifically, at the experimental set, where the non-acclimatized to LCFA inoculum was used (Fig. 1a), the addition of LCFA at a concentration of 3 g/L-reactor, resulted in a decrease of the methane yield by 95% (day 8), while the corresponding maximum decrease by adding 2 g/L-reactor was 67% (day 7). Similarly, at the experimental set that the inoculum used was previously exposed to LCFA (Fig. 1b) the doses of 3 g/L-reactor and 2 g/L-reactor lowered the methane yield by 62% and 31%, respectively. The present results are in accordance with a previous study, which reported a temporary cease of the biogas production at LCFA concentrations over 1 g/L during the thermophilic anaerobic digestion of manure^2^.

Moreover, our data validated previous findings stating that the inhibition caused by LCFA is a reversible phenomenon in the range of specific LCFA content between 1 and 5 g COD/gVSS^16^. In fact, the upset in the methane production at both experimental sets was depicted as an oscillation, starting with a dramatic drop of the methane yield shortly after the LCFA injection and followed by a time limited increase, lasting only for some days, till the system gradually returned to the initial conditions. However, it was shown that the inhibitory effect from the LCFA pulse was maintained at a lower extent in the treatment with the acclimatized to LCFA inoculum compared to the non-acclimatized one (Fig. 1). Indeed, the methane yield of the treatment with the non-acclimatized inoculum presented a very distinct perturbation (Fig. 1a) and after the initial decrease (days 5–8), there was a sudden high peak in the methane productivity (day 9). On the contrary, in the experimental set that acclimatized inoculum was used, the organic matter was degraded at a smoother rate and therefore the oscillation fluctuated in a narrower range of methane yield. This result is in agreement with a previous study reporting that inocula previously exposed to lipids accelerate the AD of lipid rich substrates^17^. As it will be further discussed, this was attributed to the more specialized microbial consortium that is populating the acclimatized to LCFA inoculum. The shock pulse resulted in a rapid accumulation of LCFA, the degradation of which is difficult to be accomplished, unless there is abundance of specific microorganisms that can process fatty acid metabolism. Finally, during period III, in both experimental sets all the reactors managed to recover to initial conditions having an average methane yield equal to 265 mL CH_4_/gVS.

**Figure 2:**
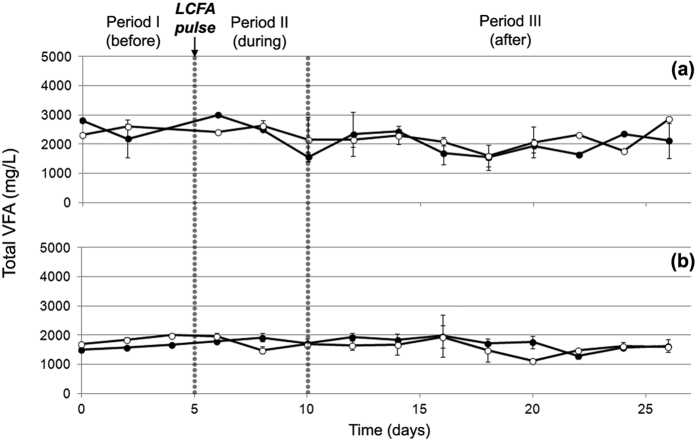
Total Volatile Fatty Acids concentration in the reactors that non-acclimatized (**a**) or acclimatized (**b**) to LCFA inoculum was used. During Period II, LCFA was injected as a single pulse to each reactor at concentrations of (●) 2 g/L-reactor or (○) 3 g/L-reactor.

An interesting finding was related with the Volatile Fatty Acids, which are the intermediate compounds of the AD process. The concentration of the total VFA remained stable at all the experimental periods and did not present any significant fluctuation even at the treatments that were subjected to higher LCFA pulse (Fig. 2). The statistical analysis (GLM) verified that there was no distinct correlation between the concentration of the total VFA and the experimental periods (p > 0.05). This is in accordance with a previous study, in which the addition of lipids as a single pulse did not lead to a VFA and pH response in thermophilic reactors treating cattle manure^18^. Nevertheless, it was remarkably found that the profile of the individual VFAs and specifically the acetate to propionate ratio (Ac/Pr ratio) has been reversed shortly after the injection of the LCFA pulse (Periods II and III) compared to the initial experimental period (Period I). More specifically, in the treatments that non-acclimatized inoculum was used, the Ac/Pr ratio at Period I was on average equal to 6, while at the end of the Period III the corresponding ratio decreased to 0.3 for all the reactors independently from the injected concentration of LCFA. Similarly, at the treatments that acclimatized to LCFA inoculum was used, the Ac/Pr ratio at Period I was on average 4.7, while at the end of the Period III the corresponding ratio decreased to 0.5 and 0.4 for the reactors that were subjected to 2 g LCFA/L-reactor and 3 g LCFA/L-reactor, respectively. The statistical analysis (GLM) showed that the property of the inocula (i.e. non-acclimatized or acclimatized to LCFA) and the experimental periods (before, during and after the LCFA pulse) are strongly correlated with the concentration of acetate, propionate and consequently with the Ac/Pr ratio (p < 0.05). Therefore, it can be extracted that the process disturbance due to the LCFA pulse was reflected specifically to individual VFAs, while by inspecting exclusively the concentration of the total VFA, the upset of the process cannot be detected. The results from the present work confirm the outcomes of a previous study suggesting that acetate, propionate and biogas production could be the state indicators for monitoring the process performance in biogas reactors^18^.

### Metagenomic assessment of the microbial community

**Figure 3:**
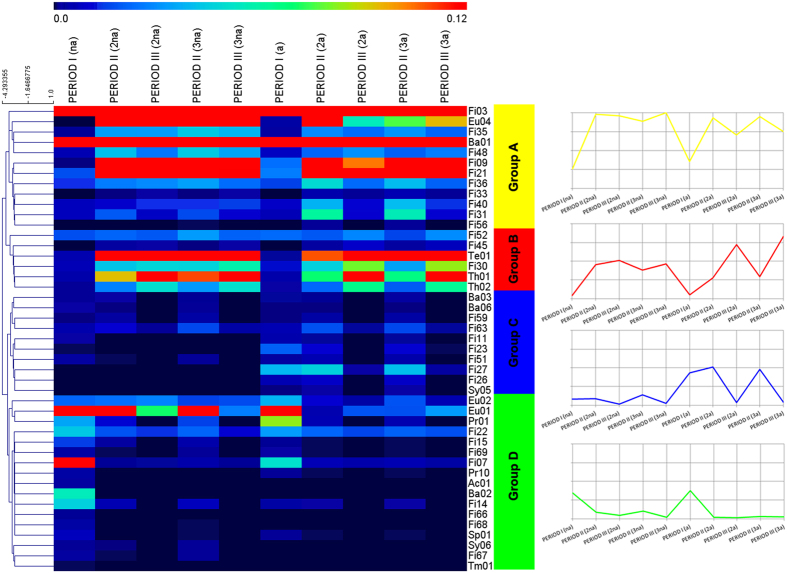
Heat map of relative abundance of the 45 Genome Bins. Correspondence between colors and relative abundance is reported in the scale at the top of each panel. The GBs could be classified into four groups as their abundance; A) increased during the LCFA pulse, B) increased during and after the LCFA pulse, C) decreased after the LCFA pulse, and D) decreased during and after the LCFA pulse. The graphs on the right illustrate the average abundance of each category.

In the present study, the shifts of the microbial dynamicity could be followed thanks to the high throughput shotgun sequencing. By aligning the reads on the metagenomic assembly, it was possible to determine the modifications in the abundance of the scaffolds across all the environmental conditions (periods). In order to ensure the analysis of the low abundant members of the microbial community, more than 16 million sequences have been obtained per each sample (Table S1). Consequently, this analysis did not rely on nucleotide sequences deposited in public databases as the scaffolds were clustered and associated to specific microbial genomes, which from now on are called Genome Bins (GB)^19^. The current version of the biogas microbial community used in the study covers 106 GBs, 45 of them presenting a distinct abundance change in response to the LCFA pulse (Fig. 3). Table S2 presents the average abundance of the 45 GBs during all the experimental periods. Thus, the present study was focused on the behavior of these specific GBs that, as it will be further discussed, were either directly involved or severely affected by the shock load induced by single pulses of unsaturated LCFA.

### Profiling of the microbial community dynamics in response to the LCFA pulse

The 45 GBs presenting a distinct dynamicity in response to the LCFA pulse could be classified into four groups as their abundance; (A) increased during the LCFA pulse (Period II), (B) increased during and after the LCFA pulse (Periods II and III), (C) decreased after the LCFA pulse (Period III), and (D) decreased during and after the LCFA pulse (Periods II and III). The initial hypothesis was that the abundance of the GBs (and therefore their classification into groups) would be mainly determined by their gene content, which obviously is the main determinant of their phenotypic properties. Indeed, the hypergeometric analysis (Dataset S1) showed that only the GBs that were positively affected by the LCFA pulse, and thus were classified to groups A and B, encode numerous genes for the metabolic pathways of “bacterial chemotaxis” and “flagellar assembly”. It is well known that these metabolic pathways are interdependent; the chemotaxis strategy of bacteria actively modulates their movement in the direction of chemoattractants by inducing clockwise rotation of flagellar motors^20^. In the current study, the Na-Oleate injected to the reactors can act as chemoattractant for the microbial species that are able to perceive and metabolize this compound. As previously mentioned, LCFAs are amphiphilic substances possessing both hydrophobic and hydrophilic ends^21^. Therefore, Na-Oleate could lead to the creation of monolayers at the liquid/air or liquid/surface interfaces of the reactors, regardless of the mixing provision, due to the structural positioning of its carboxylic ends^22^. The high fraction of genes involved in the “bacterial chemotaxis” and “flagellar assembly” pathways allows the GBs to move towards the surface of the reactor and thus to degrade the LCFA. Remarkably, metabolic pathways obviously correlated with the LCFA intervention such as “fatty acid degradation” and “stress response”, which were expected to determine the classification of the GBs, did not present distinct differences in terms of gene content among the groups. This indicates that the degradation of fatty acids is a complex process, in which microorganisms having different functional roles cooperate in order to deal with the unfavorable energetics of the conversion processes. Thus, it would not be possible to identify that all the GBs belonging to one group encode the proteins specifically for the “fatty acid degradation”.

The classification of the GBs into each group category and their change in abundance are illustrated in Fig. 3. It was clearly demonstrated that during the LCFA pulse there was an increase in the abundance of a specific bacterial GB assigned to *Syntrophomonas* genus (Fi09). It is well known that methanogens are able to grow in syntrophic association with *Syntrophomonas* sp.^12^,^14^ and sulphate reducing bacteria (SRB)^12^. The metagenomic analysis revealed that the putative syntrophic interaction of Eu04 was served by the GBs Fi09 and/or Fi56, which also presented a concomitant increase in their abundances during the LCFA pulse. Fi56 was assigned to the class *Clostridia* and according to RAST database it was related to the SRB *Desulfotomaculum*. The statistical analysis (GLM) indicated that the abundance of Fi56 was significantly and directly correlated with the methane production (p < 0.01). Moreover, Eu04, which was the only archaeon that was found to be positively affected by the LCFA pulse, was taxonomically assigned to *Methanosarcina* genus. The relative abundance of Eu04 was found to be significantly inversely correlated with acetate concentration (i.e. the more the abundance of this microbe increase, the more the concentration of acetate decrease). The co-existence of *Syntrophomonas* sp. and *Methanosarcina* sp. are in complete agreement with a previous study^14^, in which it was found that during the anaerobic degradation of LCFA, the predominant microbes presented close phylogenetic affinity to *Syntrophomonas* and a single predominant archaeon was related with the genus *Methanosarcina*. In order to further justify the role of Fi09 into the fatty acid degradation, its gene content was studied considering relevant KEGG pathways. The results showed that indeed Fi09 encodes high number of enzymes involved in the β-oxidation pathway. In the microbial community there was also another GB having a high number of genes in this specific category that was assigned to *Syntrophothermus* sp. (Fi07). Nevertheless, Fi07 was surprisingly classified to the group of microbes that decrease its abundance during and after the LCFA pulse (Group D). The low relative abundance of *Syntrophothermus* during the degradation of oleic acid was also reported by a recent study performed using a phylogenetic marker gene^23^. Therefore it was impossible to predict the functional attributes of this microorganism due to the absence of the genome sequence. Potential reasons for this peculiar behavior of *Syntrophothermus* sp. could be that either its genes involved in β-oxidation pathway undergo a different transcriptional activation, or it lacks genes involved in other metabolic pathways supporting the fatty acid degradation. Indeed, a very recent metatranscriptomic study verified that *Syntrophothermus* sp. (Fi07) presented numerous down-regulated genes, while *Syntrophomonas* sp. (Fi09) increased its transcriptional activity after LCFA exposure^24^. Using the SEED annotation subsystem, it was found that a major difference between the *Syntrophomonas* sp. (Fi09) and the *Syntrophothermus* sp. (Fi07) was that the latter one has relevant lower number of genes involved in the subsystem “Cofactors, Vitamins, Prosthetic Groups, Pigments”, and specifically no genes belonging to “Quinone cofactors” (Figure S1). As it will be further discussed in the current manuscript, some quinones are pivotal for the successful fatty acid degradation, as they are responsible for the interspecies electron transfer.

Moreover, it was found that 6 GBs were extremely sensitive to the presence of fatty acids. These were assigned to *Actinomycetales* sp. (Ac01), *Rikenellaceae* sp. (Ba02), *Clostridiales* sp. (Fi66, Fi67 and Fi68) and finally Tm01 belonging to the candidate phylum *TM7*. The statistical analysis (GLM) revealed that the abundance of the specific GBs was strongly correlated and proportionally related to butyrate and acetate concentration (p < 0.01) (Dataset S2). Therefore, as the acetate concentration remained at low levels till the end of Period III in both experimental sets (i.e. with or without acclimatized to LCFA inoculum), the relative abundance of the GBs was decreased, indicating no recovery from the inhibitory effect of LCFA. This is in agreement with the previously discussed argument that acetate serves as good state indicator for the process imbalance. A possible explanation for the sensitivity of these GBs to LCFA could be given considering the SEED annotation; Ac01, Ba02, Fi66, Fi67, Fi68 contain high number of genes involved in the carbohydrate utilization, and Fi66 has additionally high number of genes involved in the protein metabolism. Finally, all these GBs contained few or no genes belonging to the SEED subcategory “Fatty Acids”. Therefore, it could be suggested that these GBs are able to metabolize other substances than LCFA or are not specialized and a subsequent exposure to LCFA inhibits their functional properties.

Finally, concerning the archaeal community, only 3 GBs were found to be affected by the LCFA pulse. The increase in abundance of Eu04 was described previously and was associated with the formation of a syntrophic community with Fi09. The other two archaeal species (Eu01 and Eu02), decreased in abundance during and after the LCFA pulse. Both of them were assigned to the *Methanoculleus* genus; however, it was found that the relative abundance of Eu01 was higher compared to the corresponding one of Eu02. It is known that *Methanoculleus* can grow syntrophically with other bacteria in order to perform syntrophic acetate oxidation (SAO)^25^. Label isotope studies found that both acetoclastic methanogens and syntrophic acetate oxidizers were active in the methanogenic LCFA degrading microcosms^26^. Nevertheless, an enrichment of known thermotolerant or thermophilic SAO bacteria (e.g. *Tepidanaerobacter* sp. and *Thermoacetogenium* sp.) previously found to efficiently cooperate with *Methanoculleus* sp.^27^,^28^ were not detected in the current research and therefore it is suggested that this is the reason of the decreased abundance of Eu01 and Eu02. However, as it will be discussed in the next section, it is more likely that Eu01 and Eu02 presented a collective functional behavior with *Gammaproteobacteria* sp. (Pr01), indicated by the hierarchical clustering and assignment to the same group (Group D) as shown in Fig. 3.

### Elucidation of the role of the specialized to fatty acid degradation microbial consortium

Several studies highlighted the significance of the specialized inoculum in order to achieve a more efficient LCFA degradation. More specifically, the smaller lag phase, the limited inhibitory effect and the overall process stability were suggested to be due to microbial community shifts towards a new consortium which is more specialized to fatty acid degradation^17^,^29^. Even at the current study, it was shown that the LCFA pulses led to a limited process disturbance at the experimental set in which the reactors contained acclimatized to LCFA inoculum. Nevertheless, in the cited literature, the microbial community changes were studied to the best by 16S rRNA gene sequencing^12^,^14^,^30^,^31^,^32^. To the best of our knowledge, this is the first study going beyond the functional properties of GBs providing an initial deep understanding of the importance of inoculation.

The initial hypothesis was that the specialization of the inoculum should be attributed to the existence of a core microbial group which designates the LCFA exposure as the most favorable environment to grow. In the current research, we hypothesized that the acclimatized inoculum should contain high abundance of GBs with positive behavior towards LCFA, only during the shock pulse (Group A). Vice versa, according to the hypothesis, the GBs that were found to be severely inhibited during the shock pulse (Group D) should be more abundant in the non-acclimatized inoculum.

**Figure 4:**
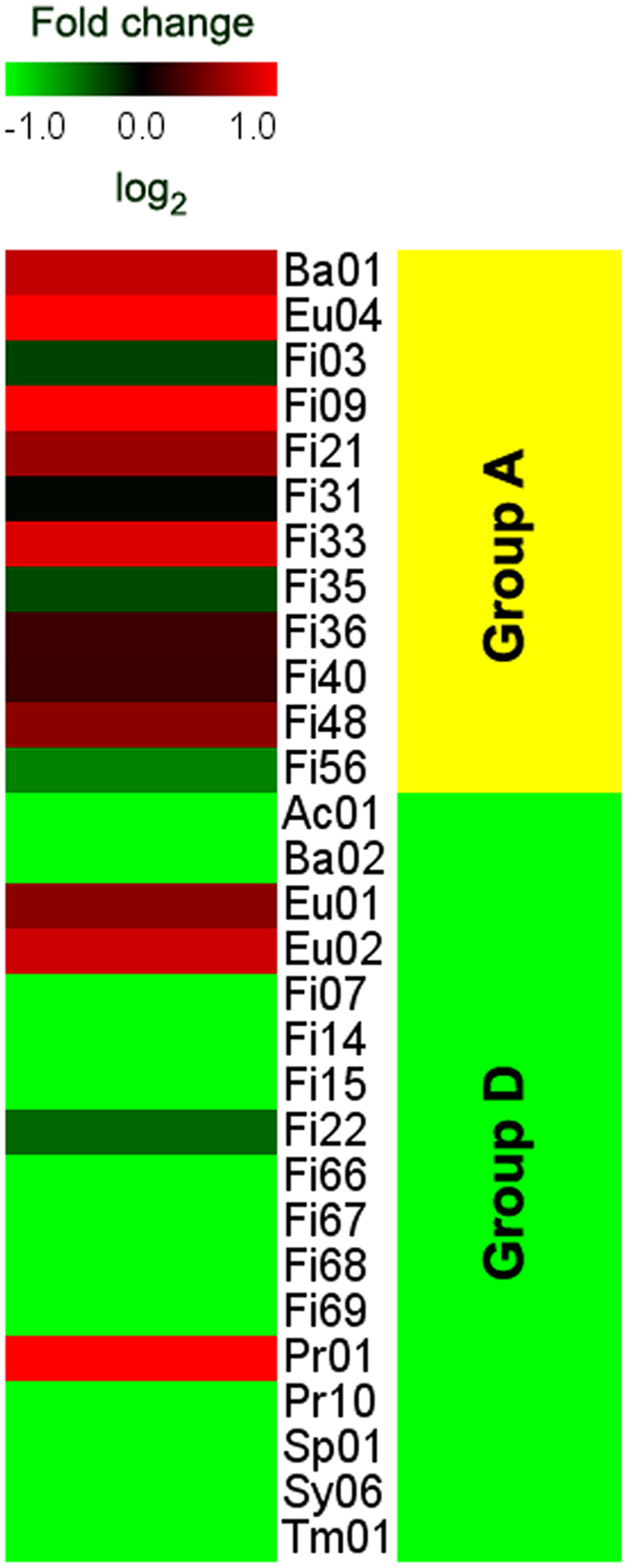
Heat map representing the relative abundance folds change of specific Genome Bins present in the non-acclimatized and acclimatized to Long Chain Fatty Acid inocula. The fold change is represented in red for higher abundance in the acclimatized inoculum and in green for higher abundance in the non-acclimatized inoculum. The GBs in each group are given in alphabetical order.

The changes in the abundance ratio of the microbial groups A and D in the non-acclimatized and acclimatized to LCFA inocula are illustrated in Fig. 4. The results validated the initial hypothesis showing that the vast majority of the GBs belonging to Group A were more enriched in the inoculum that was acclimatized to LCFA. On contrary, the GBs that were negatively affected by the LCFA exposure were more abundant in the non-acclimatized inoculum. More specifically, the major LCFA degrading bacterium Fi09 and its syntrophic methanogen Eu04 were significantly more enriched in the acclimatized inoculum, demonstrating that this is the main reason that led to increased LCFA degradation efficiency in this experimental set.

An interesting finding was related to the *Gamaproteobacteria* sp. (Pr01) and the two archaeal GBs belonging to *Methanoculleus* genus (Eu01 and Eu02). Despite that all of them were more abundant in the acclimatized inoculum (Fig. 4), they were negatively affected once the LCFA was injected into the reactors (Fig. 3). The hierarchical clustering of these GBs was extremely close, revealing a similar behavior towards the reactor interventions (Fig. 3). Focusing on the gene content of Pr01, a possible explanation for this finding could be ascribed to the quinone-mediated interspecies electron transfer. It is well known that the interspecies electron transfer is a fundamental feature in syntrophic communities between anaerobic bacteria and archaea^33^,^34^. To maintain the redox reactions of LCFA degradation at sufficiently exergonic levels, syntrophic bacteria deposit electrons onto hydrogen, which will be in turn utilized by methanogens to produce methane. Quinones, such as ubiquinone and menaquinone, function as the electron carrier between membranes complexes involved in oxidation of substrate-derived metabolites^13^. From the SEED viewer, it was found that Pr01 has a high number of genes (i.e. 20 feature counts) involved in ubiquinone biosynthesis, while *Syntrophomonas* sp. Fi09 has 11 genes involved in menaquinone biosynthesis. Nevertheless, menaquinone is preferably used by the anaerobes instead of ubiquinone to link primary dehydrogenases with terminal reductases^35^. This is due to the lower redox potential of menaquinone compared to ubiquinone^36^, allowing a more energetically favorable electron transfer to acceptors such as fumarate.

To summarize the outcomes of the present work, it was demonstrated that the toxicity caused by single LCFA pulse, even at high concentrations, is indeed upsetting the process stability, however, the inhibition is not fatally detrimental for the microbial community as the process can be recovered. The inhibition is detectable by monitoring specific biochemical parameters such as biogas production or the concentration levels of acetate and propionate. Moreover, it was demonstrated that a previously exposed to LCFA inoculum is more efficient in the degradation process of LCFA due to the specialization of the microbial consortium. By performing high throughput shotgun sequencing it was proven that the variations in abundance of the GBs can be determined with high accuracy and also connections can be established between the behavior and the genome properties. Despite the microbial species were at best taxonomically assigned at genus level, the genome properties allowed the comprehension of the functional behavior in response to the LCFA pulse. In biogas reactors that were subjected to LCFA shock loads the microorganisms were classified into four distinct groups based on their abundance shifts. By analyzing the gene content, it was interestingly found that only the microbes responding positively to the LCFA pulse could encode proteins related to “chemotaxis” and “flagellar assembly”, while the corresponding genes were totally absent from the microbes that were inhibited by the LCFA exposure. Finally, the syntrophic interactions found between *Syntrophomonas* sp. together with *Methanosarcina* sp. were possibly assigned to the quinone-electron transfer, which is a fundamental feature for maintaining the redox reactions of LCFA degradation at exergonic levels. The current research is the pioneer in analyzing the metagenome of biogas reactors subjected to LCFA shock loads. The findings will serve as base for further investigations developing strategies for optimization of the LCFA anaerobic degradation processes.

## Methods

### Influent feedstock and inoculum

Raw cattle manure used in experiments was received in one batch from Hashøj biogas plant, Denmark. After arrival, the manure was mixed thoroughly, shredded and sieved (2 mm) to separate large particles and stored at −20 °C. The frozen manure was thawed at 4 °C for 2–3 days before use. In order to minimize the effect of the indigenous microbial content of the influent manure, and isolate the observations of the microbial community changes due to the LCFA loads, the thawed manure was sterilized using an autoclave (121 °C for 20 min). The manure had a pH of 8.21 ± 0.01, total solids (TS) and volatile solids (VS) content of 37.3 ± 0.3 and 28.6 ± 0.2 g/L, respectively. The total Kjeldahl Nitrogen (TKN) and ammonium Nitrogen were 2.85 ± 0.15 and 1.62 ± 0.09 g-N/L, respectively. The concentration of total Volatile fatty acids (VFA) in raw manure was 2.69 ± 0.1 g/L. The inoculum used in the first experiment was obtained from replicate Continuous Stirred Tank Reactors (CSTR) fed exclusively with cattle manure. During the second experiment, the inoculum used was obtained from replicate CSTRs that were fed with cattle manure and supplemental amounts of Long Chain Fatty Acids (i.e. Na-Oleate, at concentration of 12 g/L feed). It should be mentioned that the influent manure of all the CSTRs were obtained from the same batch of Hashøj biogas plant, as previously described. Moreover, the inocula were obtained during the steady state periods of the replicate reactors.

### Reactor operation

Each experimental set was performed in 4 CSTRs (i.e. two biological replicates per treatment) with working volume of 0.3 L. The reactors were placed in a shaking incubator to ensure continuous mixing (stirring speed 200 rpm) and to maintain steady temperature conditions (54 °C). Initially, all the reactors were inoculated with non-acclimatized (1^st^ Experimental set) or acclimatized (2^nd^ Experimental set) to LCFA degradation thermophilic inoculum. The feeding was provided manually once every two days. The Hydraulic Retention Time (HRT) of all reactors was kept constant at 15 days. Each experimental set was divided in three periods; (a) before the LCFA shock load (Period I), (b) during the shock (Period II), which lasted from the time that the LCFA was injected to the reactors till the time that the methane production started to return to the same levels as in Period I, and (c) after the shock, which resembled the new steady state condition (Period III). The Organic Loading Rate (OLR) of the reactors during period I and III was 1.9 g VS/(L-reactor·day). The LCFA shock load was achieved by injecting as a single pulse Na-Oleate at concentrations of 2 g/L-reactor and 3 g/L-reactor.

### Analytical methods

The concentration of Total solids (TS), volatile solids (VS), pH, total Kjeldahl nitrogen (TKN) and ammonium nitrogen were measured according to APHA standard methods for the examination of water and wastewater^37^. The methane production in the reactors was measured using a gas-chromatograph (Shimadzu GC-14A, Tokyo-Japan) equipped with a molecular sieve column (2 m, 5 mm OD, 2.6 mm ID) packed with Porapak Q 80/100 mesh (Supelco, Bellefonte, PA, USA), and with a flame ionization detector (FID), as described by Kougias *et al*.^22^. The determination of the total and individual Volatile fatty acids (VFAs) analysis was performed using a gas chromatograph (Shimadzu GC-2010, Kyoto, Japan), equipped with a flame ionization detector (FID) as previously described^38^. Methane production, pH and Volatile Fatty Acid (VFA) concentration were measured once every two days. All the determinations were performed in triplicate.

### Metagenomic analysis

Genomic DNA was extracted using 15 ml of reactor’s liquid sample with Mo Bio PowerSoil DNA Isolation Kit (Mo Bio Laboratories, Inc., Carlsbad, CA). An initial step of filtration was performed as described by Bassani *et al*.^39^, in order to remove the remaining plant residues from the samples. The filtered samples were centrifuged at 2500 g for 10 min and the supernatant was discharged recovering 2 g of pellet. NanoDrop (Thermo Fisher Scientific, Waltham, MA) and Qubit fluorometer (Life Technologies, Carlsbad, CA) were used to determine the quantity and quality of the extracted DNA. Metagenome sequence was performed by the Ramaciotti Centre for Genomics (UNSW, Sydney, Australia), using Illumina HiSeq 2500 (Illumina, San Diego, CA) sequencing technology and Nextera XT kit (Illumina, San Diego, CA) for library preparation (2 × 150 bp). Further information about the extracted Genome Bins is available at the genome database www.biogasmicrobiome.com. Reads in FASTQ format were quality-filtered and the adaptors were removed using Trimmomatic software^40^. Overlapped paired-ends were merged using Flash^41^ with standard parameters, except from the maximum overlap parameter, which was set to 150. Gene finding and annotation and binning was performed following the strategy developed by Campanaro *et al*.^19^. Taxonomy was determined using Phylophlan^42^ and further refined using Phylopythia software^43^. KEGG functional analysis was refined using GhostKOALA (http://www.kegg.jp/ghostkoala/). Reads were aligned on scaffolds larger than 500 bp with Bowtie2 software^44^ and scaffold coverage was determined with the genome cov software of the bedtools package^45^. Coverage was normalized considering the number of aligned reads and using the sample with the lower number as a reference. Finally, heat maps representing the relative abundance and the folds change of microorganisms were drawn using Multiexperiment viewer (MeV)^46^.

### Statistical analysis

Statistical analyses on data have been performed using a general linear model (GLM Procedure, SAS Institute, 2009) considering the methane yield, the total VFA or the individual VFAs as traits, and the following fixed effects; (a) the property of the inoculum, as being acclimatized or not (2 levels), (b) the recording time point, which could be the shock event (Period II) or two fixed periods (~5 days) designating the steady state periods before (Period I) or after (Period III) the shock (3 levels), (c) the concentration of LCFA in each injection pulse, and (d) the abundance of each microorganism at the time of recording as linear covariate. Data were analyzed accounting the covariate of the microbial abundance as separately included within the model to avoid problems of confounding due to the shared variance and over parameterization. The least square means for the levels of the inoculum property, recording time point, and LCFA concentration have been estimated (lsmeans statement; GLM Procedure, SAS Institute, 2009), and then compared using a Student’s t-test (t diff option, GLM Procedure, SAS Institute, 2009). The variation direction (increasing or decreasing) of the methane yield and VFA in relation to the microbial species included in each analysis has been obtained from the results of the corresponding linear regression coefficient.

Hypergeometric distribution test was performed to calculate the probability of identifying the number of genes belonging to a specific functional category in each Genome Bin^47^. The probability P of finding at least k genes belonging to a specific functional category within a group of n genes (the total number of genes of a Genome Bin) is given by the following equation


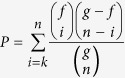


where f is the total number of genes belonging to a specific functional category considering together all the Genome Bins, g is the total number of genes determined in all the Genome Bins. Finally, we recursively repeated the calculation on KEGG pathways. All the statistical calculations were performed using the R software.

## Additional Information

**Accession codes**: Sequence data reported in this study have been submitted to the National Center for Biotechnology Information as part of the BioProject PRJNA283298. The raw sequence data were deposited at the Sequence Read Archive under the accession number SRP058179 (Table S1) and Whole Genome Shotgun projects were deposited at DDBJ/EMBL/GenBank under the accession LFRM00000000-LFTS00000000. The versions described in this paper are the first version LFRM01000000-LFTS01000000.

**How to cite this article**: Kougias, P. G. *et al*. Dynamic functional characterization and phylogenetic changes due to Long Chain Fatty Acids pulses in biogas reactors. *Sci. Rep.* **6**, 28810; doi: 10.1038/srep28810 (2016).

## Supplementary Material

Supplementary Information

Supplementary Dataset 1

Supplementary Dataset 2
