# Supplementary material for: Dynamic functional characterization and phylogenetic changes due to Long Chain Fatty Acids pulses in biogas reactors
Source: Sci Rep. 2016 Jun 29;6:28810. doi: 10.1038/srep28810 (PMC4926282; doi:10.1038/srep28810)
Supplement: Supplementary Information [file srep28810-s1.pdf]

Dynamic functional characterization and phylogenetic changes due to Long Chain Fatty Acids pulses in biogas reactors

Panagiotis G. Kougias[1,*,+], Laura Treu[1,+], Stefano Campanaro[2,+], Xinyu Zhu[1], Irini Angelidaki[1]

[1]Department of Environmental Engineering, Technical University of Denmark, Kgs. Lyngby, Denmark

[2]Department of Biology, University of Padova, Via U. Bassi 58/b, 35121, Padova Italy

* panak@env.dtu.dk

[+]These authors contributed equally to this work.

Supporting information includes:

Supplementary Table S1 and S2

Supplementary Figure S1

Supplementary Datasets S1 and S2

Supplementary Table S1. Results from the sequencing

SAMPLE NAME	SRA ACCESION NUMBER	FILTERED READS	% READS ALIGNED
PHASE1 NA-I	SRS933560	53928985	68.27%
PHASE1 NA-II	SRS933567	56397104	57.03%
SHOCK2NA-I	SRS933904	47344505	64.81%
SHOCK2NA-II	SRS933910	18623671	76.57%
SHOCK3NA-I	SRS977279	44493386	66.92%
SHOCK3NA-II	SRS977278	16941503	78.18%
STEADY2NA-I	SRS933912	39312798	66.36%
STEADY2NA-II	SRS977280	27516536	82.09%
STEADY3NA-I	SRS977277	39594102	66.61%
STEADY3NA-II	SRS977276	35220695	78.23%
PHASE1 A-I	SRS933582	57677194	70.40%
PHASE1 A-II	SRS933583	56150684	70.04%
SHOCK2A-I	SRS977273	39194561	67.64%
SHOCK2A-II	SRS977274	37263499	64.12%
SHOCK3A-I	SRS977271	36430494	69.15%
SHOCK3A-II	SRS977270	22525923	78.05%
STEADY2A-I	SRS977272	43827775	67.20%
STEADY2A-II	SRS977275	20953549	79.00%
STEADY3A-I	SRS977269	26672721	73.38%
STEADY3A-II	SRS977268	20849959	78.16%

Supplementary Table S2. List with the 45 Genome Bins and their average relative abundance within the experimental periods; non-acclimatised (na), acclimatised (a), LCFA shock of 2 g/L (2), LCFA shock of 3 g/L (3)

ID	Extended name	PERIOD I (na)	PERIOD II (2na)	PERIOD III (2na)	PERIOD II (3na)	PERIOD III (3na)	PERIOD I (a)	PERIOD II (2a)	PERIOD III (2a)	PERIOD II (3a)	PERIOD III (3a)
Ac01	Actinomycetales sp. DTU046	2.60	0.04	0.00	0.07	0.00	0.19	0.02	0.00	0.02	0.01
Ba01	Rikenellaceae sp. DTU001	29.31	108.43	93.84	94.95	115.15	49.88	69.77	78.56	69.69	73.60
Ba02	Rikenellaceae sp. DTU002	8.17	0.01	0.00	0.02	0.00	0.04	0.00	0.00	0.00	0.00
Ba03	Porphyromonadaceae sp. DTU003	0.49	0.63	0.02	0.99	0.03	1.13	0.98	0.01	0.84	0.02
Ba06	Porphyromonadaceae sp. DTU048	1.24	0.68	0.03	1.15	0.04	0.83	0.88	0.01	0.86	0.02
Eu01	Methanoculleus sp. DTU006	158.57	46.22	20.46	56.21	7.61	231.69	1.88	5.31	5.16	0.00
Eu02	Methanoculleus sp. DTU007	2.35	2.60	2.97	1.63	1.84	4.10	1.15	0.44	1.99	9.40
Eu04	Methanosarcina sp. DTU009	0.00	7.64	14.03	7.40	10.74	0.20	9.03	2.67	3.68	0.52
Fi03	Clostridiales sp. DTU012	18.95	14.71	30.53	21.27	29.02	15.83	27.97	26.93	27.74	4.54
Fi07	Syntrophothermus sp. DTU052	16.00	0.36	0.39	0.49	0.35	5.85	0.78	0.53	0.62	27.20
Fi09	Syntrophomonas sp. DTU018	0.06	20.04	12.23	14.23	11.25	1.43	16.24	6.21	24.37	0.46
Fi11	Clostridiales sp. DTU020	1.41	0.11	0.01	0.16	0.02	4.25	2.46	0.02	2.55	11.06
Fi14	Clostridiale sp. DTU023	6.16	0.64	0.03	0.94	0.05	0.55	0.28	0.02	0.28	0.03
Fi15	Clostridiales sp. DTU024	4.16	0.71	0.01	1.22	0.03	0.88	0.14	0.02	0.15	0.02
Fi21	Halothermothrix sp. DTU029	3.31	36.17	23.95	26.59	22.50	5.02	47.77	29.56	51.05	0.03
Fi22	Clostridia sp. DTU030	6.33	2.68	1.95	3.14	1.59	4.78	3.14	2.27	3.03	31.79
Fi23	Clostridiales sp. DTU031	0.16	0.06	0.01	0.09	0.02	6.77	3.53	0.15	3.63	2.65
Fi26	Clostridiales sp. DTU035	0.02	0.02	0.00	0.02	0.00	1.25	2.00	0.02	2.03	0.20
Fi27	Clostridiales sp. DTU036	0.00	0.00	0.00	0.01	0.00	5.19	6.04	0.26	5.41	0.03
Fi30	Clostridiales sp. DTU058	1.14	7.13	8.10	7.94	9.84	2.16	7.86	13.65	5.89	0.33
Fi31	Clostridiaceae sp. DTU059	1.54	4.18	1.75	3.73	2.16	1.51	13.26	2.23	12.14	24.11
Fi33	Clostridia sp. DTU062	0.20	1.22	2.28	1.52	2.44	0.37	6.92	2.00	6.07	14.28
Fi35	Clostridiales sp. DTU064	0.48	5.17	5.07	6.73	6.04	0.39	4.50	3.54	4.78	2.38
Fi36	Clostridia sp. DTU065	1.25	2.54	2.82	3.27	2.23	1.48	4.74	2.21	3.88	1.86

Fi40	Clostridiales sp. DTU070	0.81	1.02	1.40	1.39	1.59	0.95	4.13	1.18	4.39	3.38
Fi45	Clostridiales sp. DTU076	0.10	1.38	1.05	0.57	1.17	0.13	1.29	4.55	1.38	2.01
Fi48	Clostridiaceae sp. DTU079	0.35	2.81	1.70	3.00	2.40	0.52	1.45	2.05	1.38	1.43
Fi51	Clostridiales sp. DTU082	1.02	0.32	0.02	0.59	0.03	1.10	1.86	0.06	1.83	0.03
Fi52	Clostridiales sp. DTU083	1.49	1.82	1.62	2.78	1.80	1.95	1.62	2.40	1.32	3.72
Fi56	Clostridia sp. DTU088	0.29	0.46	0.36	0.62	0.38	0.20	1.78	0.15	1.68	1.81
Fi59	Erysipelothrix sp. DTU091	0.23	0.44	0.04	0.70	0.07	0.23	0.71	0.05	0.73	0.08
Fi63	Eubacteriaceae sp. DTU096	0.48	0.85	0.14	1.34	0.30	0.38	1.46	0.27	1.34	2.54
Fi66	Clostridiales sp. DTU099	1.48	0.13	0.00	0.20	0.00	0.12	0.04	0.00	0.04	0.19
Fi67	Clostridiales sp. DTU100	0.88	0.25	0.01	0.50	0.02	0.07	0.05	0.02	0.05	0.05
Fi68	Clostridiales sp. DTU101	1.76	0.18	0.00	0.31	0.00	0.03	0.01	0.00	0.01	0.30
Fi69	Clostridiales sp. DTU071	5.27	1.08	0.03	1.99	0.06	2.42	0.83	0.02	0.86	0.00
Pr01	Gammaproteobacteria sp. DTU037	5.64	1.85	0.01	2.46	0.01	13.38	0.87	0.01	1.05	0.03
Pr10	Alcaligenaceae sp. DTU106	2.35	0.14	0.00	0.21	0.01	0.97	0.35	0.01	0.41	0.00
Sp01	Spirochaeta sp. DTU042	1.74	0.08	0.00	0.12	0.00	0.36	0.14	0.00	0.14	0.01
Sy05	Synergistaceae sp. DTU109	0.01	0.01	0.00	0.01	0.00	0.38	0.71	0.07	0.69	0.01
Sy06	Synergistales sp. DTU110	0.80	0.55	0.06	0.84	0.10	0.11	0.14	0.01	0.14	0.00
Te01	Acholeplasmatales sp. DTU056	0.37	14.90	11.45	8.53	8.05	0.20	6.51	17.34	8.52	0.08
Th01	Thermotogaceae sp. DTU111	0.67	14.09	27.30	16.52	26.84	0.65	10.50	33.37	9.85	0.02
Th02	Thermotogales sp. DTU112	0.47	7.69	14.04	8.99	14.26	0.55	6.22	18.16	5.63	31.83
Tm01	TM7 DTU050	0.54	0.15	0.00	0.23	0.01	0.01	0.00	0.00	0.00	17.69

a) Fi07- *Syntrophothermus* sp. DTU052

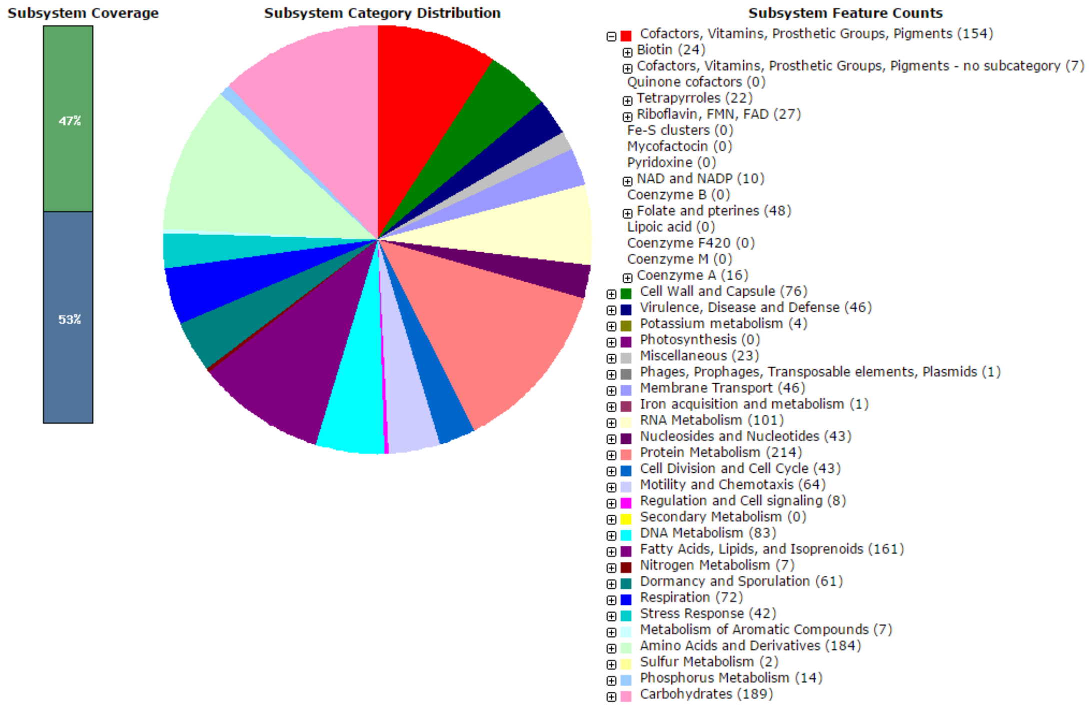

b) Fi09- *Syntrophomonas* sp. DTU018

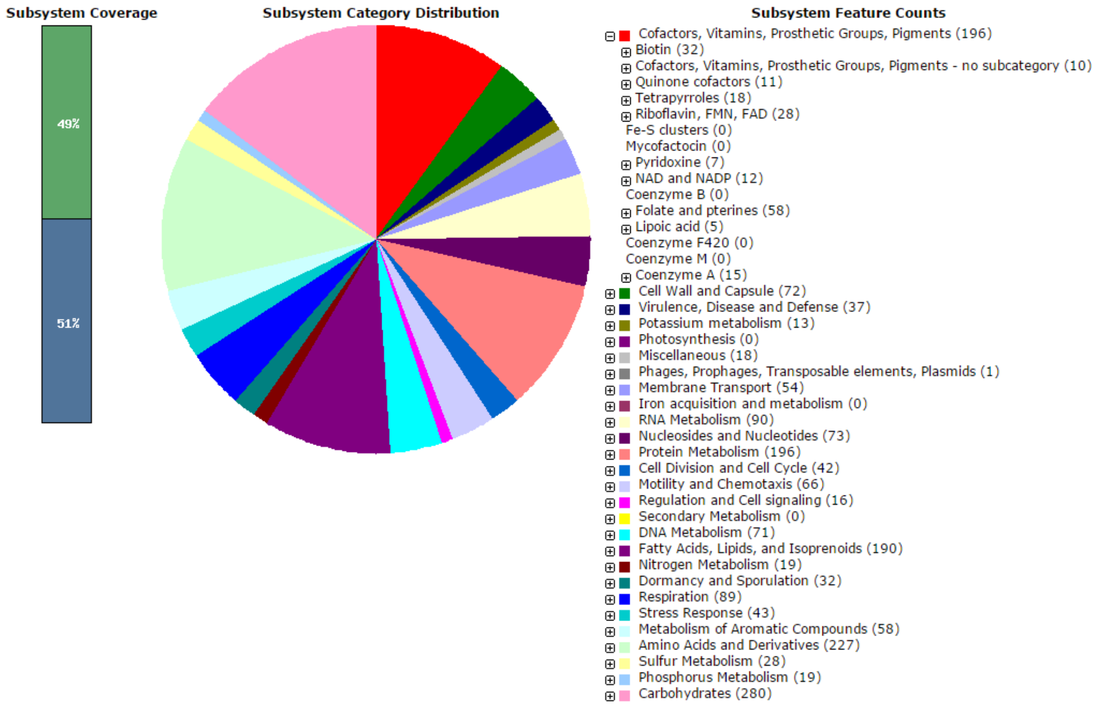

Supplementary Figure S1. SEED annotation for a) *Syntrophothermus* sp. (Fi07) and b) *Syntrophomonas* sp. (Fi09) focusing on the subsystem "Cofactors, Vitamins, Prosthetic Groups, Pigments".
